# Evaluating TabPFN: a transformer-based foundation model for explainable health insurance claim prediction

**DOI:** 10.3389/fpubh.2026.1880390

**Published:** 2026-06-26

**Authors:** Mazen Shawosh, Haseeb Nisar, Saleh Alwahaishi

**Affiliations:** 1 ISOM Department, KFUPM Business School, King Fahd University of Petroleum & Minerals (KFUPM), Dhahran, Saudi Arabia; 2 IRC for Finance and Digital Economy, King Fahd University of Petroleum & Minerals (KFUPM), Dhahran, Saudi Arabia

**Keywords:** explainable AI, foundation model, health insurance, machine learning, regression

## Abstract

**Background:**

Predictive modeling for companies involved in health insurance policies remains an active area of actuarial research as they seek to leverage Machine Learning (ML) applications to increase productivity and improve operational efficiency. Despite the success of classical machine learning models on tabular insurance datasets, they often require feature engineering and hyperparameter tuning, hence posing scalability and deployment challenges. While addressing these limitations and an identified gap in the literature, this research evaluated a next-generation Transformer-based tabular foundation model, the Tabular Prior-Data Fitted Network (TabPFN-2.5, Prior Labs, Germany), for health insurance claim prediction and benchmarked its performance against established ML models.

**Methods:**

The study utilizes a primary dataset comprising 13,904 records with 13 features, along with three additional validation datasets to assess robustness across heterogeneous data distributions. Our approach involves extensive preprocessing, including data cleaning and converting categorical features into a numerical data type. Model performance was extensively evaluated using metrics of Root Mean Squared Error (RMSE), Mean Absolute Error (MAE), and R-squared (*R*^2^).

**Results:**

The results showed that the TabPFN regression model consistently outperforms the baseline models, achieving an *R*^2^ of 0.9776 on the primary dataset and demonstrating better generalization across validation datasets. The impact of each feature on the model's prediction was evaluated using SHapley Additive exPlanations (SHAP) as an Explainable Artificial Intelligence (XAI) method, revealing smoking status, age, and body mass index as the most influential determinants.

**Conclusion:**

This research highlights the impact of TabPFN on improving predictive modeling of healthcare finances and on selecting the most suitable policies for customers.

## 1 Introduction

A health insurance policy is designed to cover or reduce the financial burden of losses resulting from various health risks. Health insurance plays a pivotal role in advancing universal health coverage, an essential component of the Sustainable Development Goals (1). It had a significant impact in underdeveloped areas, notably among the working middle-class sector, as well as on infants and people with disabilities (2). The need for medical insurance is evident from the recent COVID-19 pandemic, which underscored the importance of financial backup (3). The cost of health insurance varies from person to person because factors such as demographic profile, health status, geographic access to care, and lifestyle can significantly influence projected healthcare costs. Among other influential factors that may affect the potential cost of medical insurance are the scope of coverage, the type of plan, and the customer's age (4). This can be explained by the notion that older individuals require more frequent medical care and have higher rates of chronic conditions than younger people, thereby indicating higher insurance premiums for older age groups.

Conventionally, medical insurance companies rely heavily on human intervention, leading to operational delays, errors, and higher costs. For the smooth and effective development of insurance premium packages and for detecting health insurance fraud, machine learning (ML) models help these insurance companies with the added advantage of solving imbalanced data and improving detection efficiency (5, 6). Predictive analytics enables insurance companies to forecast future health risks for their policyholders. This allows them to tailor preventive measures for individuals, helping avoid costly medical complications down the line. Regression methods, commonly employed for these purposes, consistently yield adequate precision (7, 8). This has been demonstrated in a recent study where five different regression models were used to develop a real-time medical insurance cost price prediction system using ML (7). The most effective ML algorithm will be selected by evaluating its performance on the testing dataset. When applying ML models to health insurance datasets, it is important to verify the data structure. Most health insurance datasets are tabular, containing multiple features (9–11). Traditionally, machine learning methods for tabular datasets have been the main toolkit for applied data science practitioners (12, 13). An XGBoost model was employed to predict health insurance costs and to perform flexible imputation of missing data (14). In another study, Logistic Regression (LR) and XGB techniques were used to predict the presence of a small number of accident claims, demonstrating the superior performance of LR over XGBoost because of its interpretability and strong predictability (15). Hierarchical decision trees and other ML models have been utilized for healthcare cost predictive analytics, justifying the important role of machine learning in the healthcare sector, particularly for diagnostic applications and medical insurance cost forecasting (12, 13). However, these models have notable limitations, such as requiring extensive, dataset-specific tuning (16), overfitting of data (17), failing to quantify uncertainty without significant adjustments, and exhibiting limited generalization compared to modern foundational models (18). Contrastingly, deep learning methods have historically faced challenges with tabular data, stemming from both cross-dataset heterogeneity and the intrinsic variability of the raw data (19). A shift that addresses these problems is represented by tabular foundation models (TFMs) (20). Transformers are the preferred architectures for operating these foundation models (21–23), where attention mechanisms are utilized to capture long-range dependencies and learn complex relationships within the data (24, 25). Previously, Actuarial Transformer (AT) architecture was used together with the SHAP model to increase the auto insurance risk evaluation accuracy and its interpretability (26). FMs do not need training since they are pretrained on various synthetic tabular datasets and make predictions through in-context learning rather than gradient descent (20, 27). Instead of the time-consuming hyperparameter tuning needed for gradient-boosted trees, these models are meta-trained to provide well-calibrated predictions. They are also advantageous in low-data scenarios because of their strong generalization capabilities (28).

The objective of this research is to evaluate the performance of a new generative transformer-based foundation model, the Tabular Prior-data Fitted Network (TabPFN) (29), and to compare its performance across established classical ML models for health insurance datasets. Prior-data fitted networks like TabPFN are trained on 100 million synthetic data points to mimic Bayesian inference. These synthetic datasets replicate intricate phenomena such as missingness, non-linearity, categorical mixing, and temporal shifts, enabling TabPFN to adapt robust priors while circumventing the privacy issues associated with it. As a result of this training, TabPFN can recognize intricate patterns in tabular data and can also be used with completely different datasets without any additional effort. To create a potent tabular prediction algorithm that is fully learned from data, TabPFN leverages in-context learning (ICL) (30), an approach that has propelled the success of large language models. ICL enables exploration of a larger algorithmic space, including cases where closed-form solutions are unavailable. The innovation of TabPFN lies in moving away from the traditional machine learning approach of training on a single task. It uses meta-learning (31), structural causal inference method (32), and global attention (33) to build a general intelligent system designed for tabular data. The recent version of this model, TabPFN-2.5, not only handles the messy, heterogeneous data, including categorical features, missing values and outliers, but also scales the power of in-context learning, supporting up to 50,000 samples and 2,000 features.

Moreover, the incorporation of SHapley Additive exPlanations (SHAP) made the model transparent by revealing the feature contributions, thus giving the health insurance companies the ability to comprehend and have confidence in the model's predictions. On the other hand, our approach serves as an alternative methodology for health insurance prediction, which not only solves the major problems of past methods but also enhances prediction results and enables fair health insurance premium plans.

## 2 Methodology

### 2.1 Dataset and exploratory data analysis

The study used the medical insurance dataset (34) from the KAGGLE database, along with three other datasets for model validation. Similar types of datasets have been used previously from the same repository in earlier studies (4, 35, 36). The primary dataset was collected from individuals aged 18–64 years and contains 15,000 records along with 13 features. Data cleaning was performed to eliminate erroneous data and ensure the model's reliability thereafter. This includes identifying and removing missing and duplicates from the dataset. Categorical columns (smoking status, sex, hereditary diseases, job title, and city) were encoded via one-hot encoding and converted into a numerical data type. The data was then normalized using the StandardScaler technique.

Exploratory Data Analysis **(**EDA) was applied before applying machine learning models, as this helps us understand the data and identify potential problems or areas of interest. The relationship between the target variable and the corresponding features was explored further using data visualization tools to reveal obscured connections between features. In our dataset, the target variable was “claim”, which represents the total medical expenses (in USD) billed by the health insurance company to a policyholder, and all models were trained to predict this continuous outcome as a regression task and the features were “age”, “sex”, “body mass index (BMI)”, “hereditary diseases”, “smoking status”, “number of dependents”, “residential area”, “blood pressure”, “diabetes presence” and job title.

A correlation matrix was constructed and visualized using a heat map to enable us to identify which features are most important and have a strong impact on Insurance claims.

### 2.2 Machine learning models

We applied three tree-based methods, i.e., Random forests (37), XGBoost (38) and CatBoost (39) regression models, due to their superior performance in making predictions on tabular data (19, 40). All preprocessing steps (StandardScaler normalization, one-hot encoding of categorical variables) were wrapped into a scikit-learn Pipeline and applied only on training folds over the course of cross-validation. The scaler and encoder were fit on each training fold and applied to the corresponding validation fold, thereby preventing any form of data leakage. Their performance was evaluated using standard evaluation metrics, including the Root Mean Squared Error (RMSE), the Mean Absolute Error (MAE), and the coefficient of determination (*R*^2^). All models were developed within the same experimental framework to ensure a fair and unbiased comparison. This approach provides confidence in evaluating the behavior and effectiveness of these models without the influence of external confounding factors. The data was divided into two parts: 70% for training and 30% for testing. From the training dataset, models were built to predict medical insurance costs, while the test dataset was used to evaluate the model's performance. Additionally, we utilized the pre-trained Transformer models Tabular Prior-Data Fitted Network (TabPFN) and a train-from-scratch neural network model, FT-Transformer (Feature Tokenizer + Transformer), both specifically designed for tabular data and offering valuable insights for developing prognostic scores. For models that do not natively handle categorical variables, preprocessing was performed using a ColumnTransformer to encode string data appropriately. Each model was evaluated using 5-fold cross-validation, with *R*^2^ (coefficient of determination) as the scoring criterion. The cross-validation process was controlled for reproducibility by setting a fixed random seed (random_state = 42) and enabling data shuffling. We also performed hyperparameter optimization for the three ensemble-based regression models and optimized using randomized hyperparameter search. A RandomizedSearchCV procedure with 40 random configurations was employed to efficiently explore the hyperparameter space. For the Random Forest model, hyperparameters controlling tree depth, ensemble size, feature subsampling, and node splitting criteria were optimized. XGBoost tuning focused on boosting dynamics, tree complexity, sampling ratios, and regularization parameters, using the histogram-based tree construction method for computational efficiency. CatBoost hyperparameter optimization targeted learning rate, tree depth, regularization strength, and bagging behavior. The model performance was measured using cross_val_score, which returns the *R*^2^ scores for each fold. The mean *R*^2^ score for each model was then computed to summarize overall performance. Finally, a bar chart was generated to visualize the models based on their average *R*^2^ scores. We implemented feature importance calculation using SHAP (Shapley Additive Explanations) 14, a game-theoretic method for explaining predictions. SHAP values represent the contribution of each feature to the model's output, treating each feature as a contributor. The model produces a prediction value for each instance, and the contribution of each feature in that instance is represented by its SHAP value.

The experiments were conducted using Python 3.10.12 and specific libraries, including scikit-learn 1.4.0, XGBoost 2.0.3, CatBoost 1.2.2, SHAP 0.44.1, and PyTorch 2.1.0. The TabPFN was utilized through the tabpfn Python package (v2.0.1) without task-specific hyperparameter tuning, adhering to its in-context learning framework. To ensure reproducibility, global random seeds were set before all experiments, alongside a predefined random state for cross-validation. The experiments took place on a system outfitted with an NVIDIA A100 40 GB GPU and 64 GB RAM. The complete analysis code will be publicly accessible via GitHub upon acceptance of this manuscript. At inference time, TabPFN-2.5 utilizes the entire labeled training set alongside unlabelled test instances in a single forward pass through the pre-trained transformer, employing in-context learning without gradient updates or weight modifications. The model operates using its default TabPFNRegressor settings, with no fine-tuning or prompt engineering, consistent with its zero-shot tabular prediction capabilities.

## 3 Results

### 3.1 Datasets acquisition and EDA

The dataset used for model construction contains 15,000 samples with 13 attributes/features as described in Table 1 and Supplementary Table 1. Data preprocessing, an essential part of downstream analysis, was performed, including checking for missing and duplicate values. There were 396 and 956 missing values in column Age and BMI, respectively, and 1096. Since the data in both columns have skewness between the range −0.5 to +0.5, they are symmetrical and proceed with filling null values with the mean. In addition, we found 1,096 duplicated values, which were removed from the raw data, resulting in 13,904 rows.

**Table 1:** Description of the primary dataset used for building machine learning models.

Type	Name	Description	Data type
Features	Age	Customers' age in years	Numerical
	Sex	Gender status of customer (Male/Female)	Categorical → Numerical
	Weight	Weight of customer in (kg)	Numerical
	BMI	Body mass index	Numerical
	Hereditary diseases	Presence or absence of any disease	Categorical → Numerical
	Dependents	Number of dependents of customer	Numerical
	Smoker	Smoking status (Yes/No)	Categorical → Numerical
	City	City where the customer lives	Categorical → Numerical
	Blood pressure	Customer blood pressure status	Numerical
	Diabetes	Diabetes status of customer (diabetic/non-diabetic)	Categorical → Numerical
	Regular exercise	Customer regular exercise status (Yes/No)	Categorical → Numerical
	Job title	Job title of the customer	Categorical → Numerical
Target variable	Claim	Insurance premium price	Numerical

We employed one-hot encoding to convert the categorical variable into a numerical data type. The other three datasets used for validation include 1,338 samples and seven features, 1,332 samples with 11 features, and 986 samples with 11 features.

**Figure 1:**
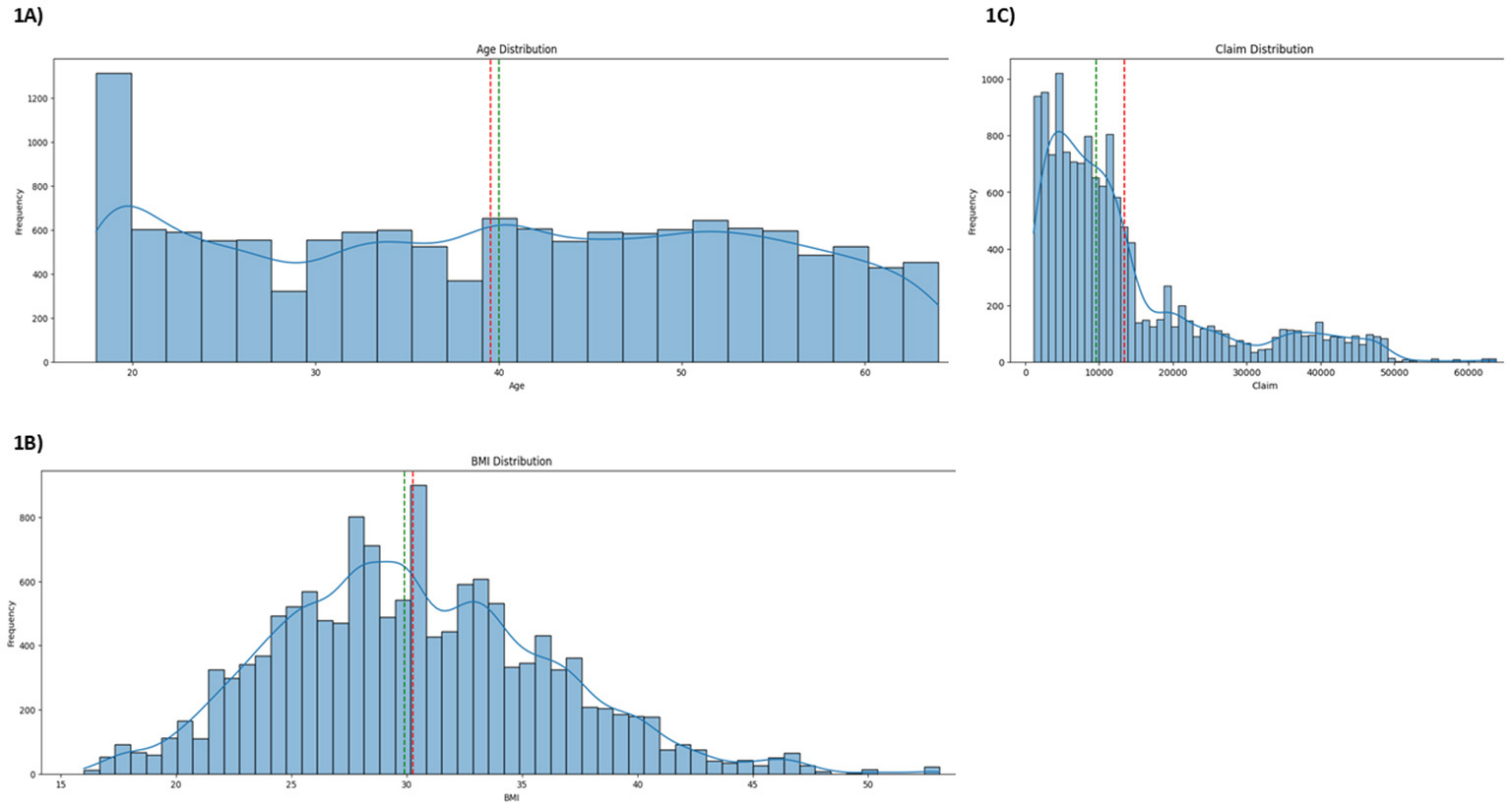
Distribution of demographic and clinical characteristics among study participants. **(A)** Age distribution shows a uniform spread across adulthood. **(B)** BMI distribution exhibiting a normal shape, centered around the overweight range (BMI ≈ 30 kg/m^2^), with most participants between 25–35 kg/m^2^. **(C)** The distribution of charges demonstrates a right-skewed pattern with most participants incurring low to moderate expenses.

The EDA analysis revealed different patterns among the dataset's variables. The age distribution is uniform across adulthood, with a skewness value close to 0, with an average age of the clients of around 40 years, with the youngest being 18 years old and the oldest being 64 years old. This shows a good representation of various adult age groups without significant age-related bias (Figure 1A). The distribution of BMI is close to normal, centered around BMI levels of approximately 30 kg/m^2^. Most participants have a BMI between 25 and 35 kg/m^2^, with fewer at the extremes. This indicates that the group mostly includes overweight and mildly obese individuals, which is typical of many clinical populations (Figure 1B). The distribution of claims or charges was right-skewed, indicating the premium prices are more frequent at the lower end of the pricing range and less frequent at the upper end, as customers take them (Figure 1C). On average, clients have claims of around dollar 13,431. However, the clients whose claims exceed 60,000 dollars, and were considered as outliers, also existed in the data, that cause the distribution of the claims column to be right-skewed with a skewness value of 1.48.

**Figure 2:**
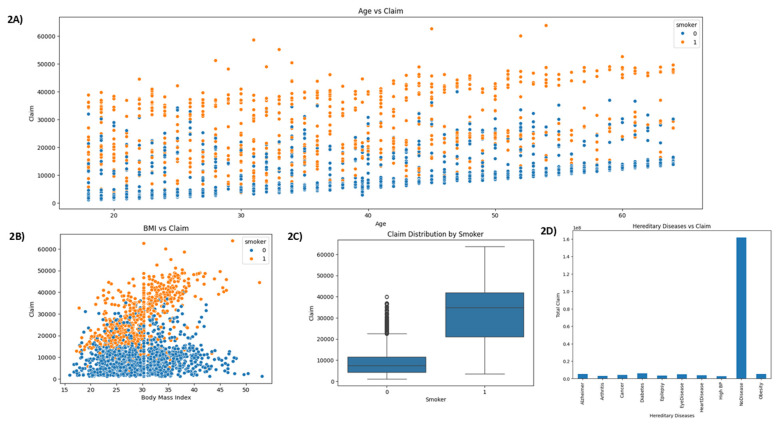
Associations between age, BMI, smoking status, and insurance claim amounts. **(A)** Age vs. claim: Insurance claims increase with age, indicating higher healthcare costs among older adults. **(B)** BMI vs. claim: Claim amounts show moderate dispersion across BMI, with higher values among overweight and obese individuals. **(C)** Smoker vs. claim: Smokers display significantly higher and more variable claim amounts compared to non-smokers, reflecting the elevated healthcare burden of smoking-related conditions. **(D)** Hereditary vs. claim: Individuals with no disease have a higher insurance claim. ^*^(0 indicates Non-smoker and 1 indicates Smoker).

The association of key demographic and behavioral factors, such as age, BMI, smoking status, and hereditary diseases with insurance claim amounts is shown in Figure 2. A positive relationship was seen between age and claim values, with older individuals requiring higher medical expenses (Figure 2A). It is also evident that the claims for smokers are significantly higher than those for non-smokers. Claim amounts also varied moderately across BMI values. Costs were slightly higher among individuals with higher BMI, suggesting a small association between excess body weight and healthcare spending (Figure 2B). The scatter plot also reveals that individuals with smoking and high bmi have higher claim amounts than non-smokers. Smoking status showed the most significant association with the health insurance claim amount. Smokers showed much higher median claim amounts and greater variability in their claims than non-smokers (Figure 2C). This was observed in the data analytics, where the total number of smokers was 11,114 clients, making up roughly 20% of all claims. Nevertheless, even though they represent a minor segment, the expense incurred by a smoker is approximately $32,102, which is considerably more than the $8,745 paid by non-smokers. The association between hereditary diseases and claim amount suggests that individuals with no disease have a significantly higher claim as compared to others (Figure 2D). These patterns suggest that aging and changeable risk factors, mainly smoking, are associated with higher healthcare costs for those insured.

**Figure 3:**
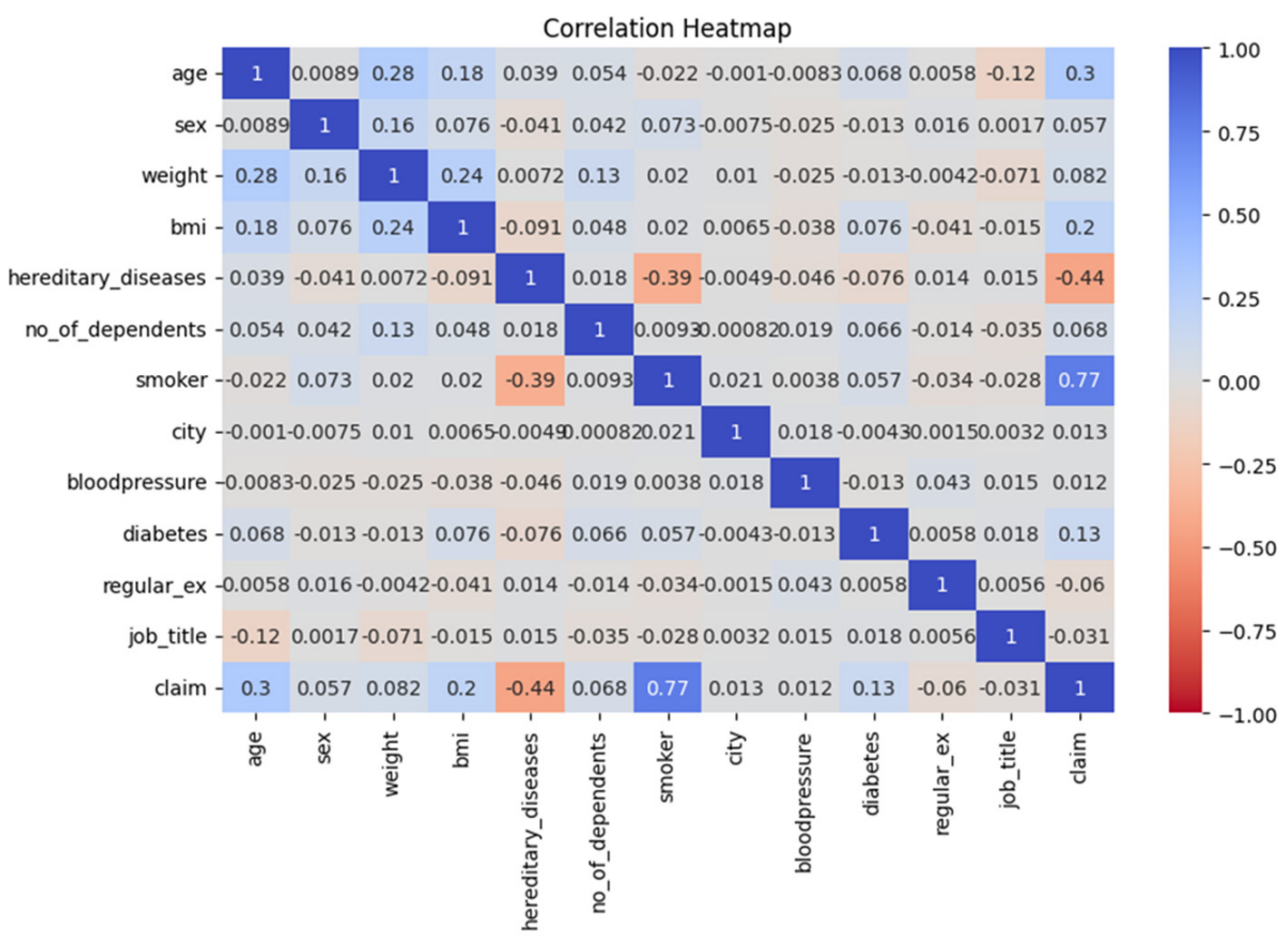
Correlation matrix of demographic, lifestyle, and clinical variables with insurance claim amounts, with a strong positive correlation between claim amounts and smoking status.

Finally, the correlation matrix was visualized to display the numerous factors that possess both strong and weak relationships with one another. The correlation matrix shows that the claim amount has the highest positive correlation with smoking status (*r* = 0.77), followed by age (*r* = 0.31), and BMI (*r* = 0.22), indicating that smoking status had the strongest correlation with healthcare costs in this dataset (Figure 3).

### 3.2 Machine learning model

The training and testing datasets were divided in proportions of 0.7 and 0.3, respectively, with 5-fold cross-validation. We compared five different machine learning models, two of which are Transformer models and three are tree-based ensemble models. Among the evaluated models, TabPFN achieved the highest predictive performance, with an *R*^2^ score of 0.9776, indicating a superior ability to capture the underlying relationships in the data. Ensemble-based methods, including Random Forest, XGBoost, and CatBoost, also performed well, with *R*^2^ values of 0.9685, 0.9656, and 0.9614, respectively. No substantial evidence of overfitting was observed, as training and testing R^2^ values remained consistent across folds.

The FTTransformer was developed utilizing the pytorch_frame library and consists of 6 transformer encoder layers with 8 attention heads and a feed-forward dimension of 512. Training was conducted with the AdamW optimizer, employing a learning rate of 1 × 10^−4^, weight decay of 1 × 10^−5^, a batch size of 256, and a maximum of 100 epochs, incorporating early stopping based on validation R^2^ to mitigate overfitting. During hyperparameter tuning, various learning rates ({1 × 10?3, 1 × 10^−4^, 1 × 10^−5^}), feed-forward dimensions ({256, 512}), and layer depths ({4, 6, 8}) were evaluated. The optimal configuration was determined through 5-fold cross-validation on the primary dataset.

**Figure 4:**
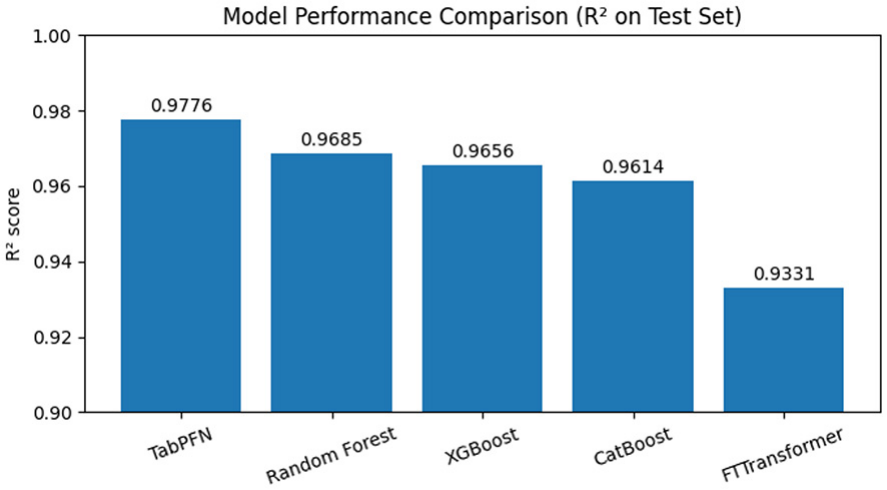
Mean *R*^2^ scores from 5-fold cross-validation comparing TabPFN, Random Forest, XGBoost, CatBoost, and FTTransformer models. Higher values indicate better variance explanation.

Contrastingly, the TabPFN consistently outperformed these tuned models without requiring any dataset-specific hyperparameter tuning. This shows the benefit of foundation-model-based in-context learning compared to traditional optimization-driven workflows. The FTTransformer model showed comparatively lower performance (*R*^2^ = 0.9331) (Figure 4). Overall, the results indicate that while all models achieved high predictive accuracy, TabPFN consistently outperformed the other approaches under cross-validation, suggesting its robustness and suitability for this prediction task. The TabPFN results were validated across three other datasets with fewer samples. Given the inherent advantage of earlier versions of TabPFN, which were specifically designed for small-to-medium-scale tabular data, the model outperformed all datasets, demonstrating its superior performance across small to large-scale datasets compared to tree-based ensemble models (Table 2).

**Table 2:** The table shows the performance of five different Machine learning algorithms with their Mean *R*^2^, Mean MAE, and Mean RMSE values across four different sample size health insurance tabular datasets.

Dataset	Number of samples	Model	Mean *R*^2^ (training)	Mean *R*^2^ (testing)	Mean MAE (training)	Mean MAE (testing)	Mean RMSE (training)	Mean RMSE (testing)
Primary dataset	13,904	TabPFN	0.999	0.9776	39.24	342.54	127.97	1,825.49
		Random forest	0.995	0.9685	154.81	428.34	780.59	2,142.27
		XGBoost	0.994	0.9656	440.76	754.18	901.57	2,238.21
		CatBoost	0.980	0.9614	902.97	1,134.84	1,673.09	2,370.55
		FTTransformer	0.952	0.9331	112.45	255.52	454.45	652.43
Validation dataset 1^†^	1,338	TabPFN	0.875	0.8821	1,703.48	2,224.19	4,260.47	4,473.79
		Random forest	0.875	0.8755	2,516.14	2,516.14	4,601.09	4,601.09
		XGBoost	0.874	0.8725	2,295.28	2,609.83	4,105.33	4,657.10
		CatBoost	0.871	0.8701	2,566.37	2,566.36	4,689.85	4,689.85
		FTTransformer	0.872	0.8754	2,013.63	2,367.82	3,932.56	4,273.50
Validation dataset 2	1,332	TabPFN	0.9999	0.9998	36.956	54.53	102.58	182.17
		Random forest	0.9999	0.9995	19.031	58.947	87.97	155.75
		XGBoost	0.9317	0.9991	883.68	130.06	1,575.55	442.46
		CatBoost	0.9999	0.9994	62.833	132.79	83.89	265.39
		FTTransformer	0.965	0.9796	657.87	1,081.58	1,254.56	1,627.30
Validation dataset 3	986	TabPFN	0.893	0.831	505.57	903.73	2,032.78	2,616.63
		Random forest	0.826	0.826	1,245.69	1,245.69	2,771.92	2,771.92
		XGBoost	0.931	0.824	883.68	1,480.07	1,575.55	2,787.30
		CatBoost	0.795	0.795	1,695.23	1,695.23	3,006.71	3,006.71
		FTTransformer	0.794	0.726	2,054.23	2,299.30	2,965.54	3,291.52

^†^Metrics for Validation Dataset 1 are rounded to highlight negligible visual variance, though computed on distinct data splits, training and testing MAE/RMSE values for Random Forest and CatBoost appear identical when rounded to 2 decimal places; values reported to 4 decimal places confirm that training and testing metrics are computed on distinct splits and are not equal.

**Figure 5:**
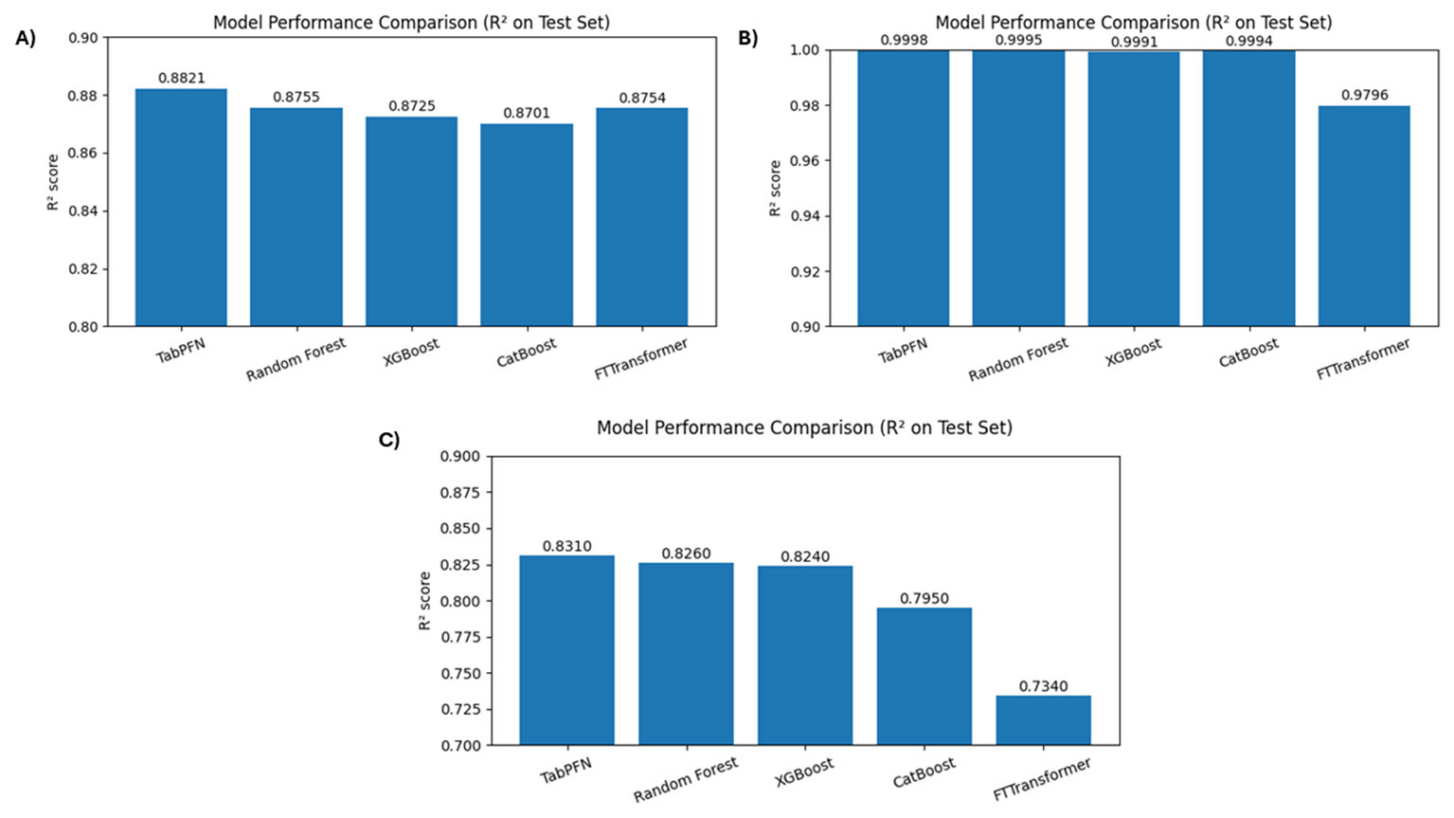
The figures show the performance of five different machine learning models. Panels **(A–C)** show the variance explained (*R*^2^) across three evaluation settings and highlight differences in model accuracy and robustness across validation datasets.

For validation dataset 1, all models demonstrated *R*^2^ values exceeding 0.8. However, TabPFN achieved the highest variance explained (*R*^2^ = 0.8821), which was followed by RandomForest (0.8775) and FTTransformer (0.8754) (Figure 5A). The model's performance improved significantly on validation dataset 2, with TabPFN achieving near-perfect variance explanation (R^2^ = 0.9998) and marginally outperforming Random Forest, XGBoost, and CatBoost (Figure 5B). The near-perfect *R*^2^ of 0.9998 on Validation Dataset 2 likely reflects the highly structured and low-noise nature of this Kaggle dataset, where a small number of dominant features account for nearly all variance in claim amounts. This performance should not be interpreted as indicative of general out-of-distribution generalizability, and we caution against over-interpreting this result. In contrast, FTTransformer exhibited a noticeable decline in performance compared to the other models, suggesting reduced generalization under this evaluation condition. Finally, TabPFN consistently outperformed all other approaches, achieving the highest *R*^2^ value of 0.831 for validation dataset 3 (Figure 5C).

Overall, these results highlight the superiority of TabPFN across all conditions evaluated, while ensemble-based methods, such as Random Forest and CatBoost, were stable but yielded slightly lower predictive accuracy. The performance of the neural-based model, FTTransformer, varied significantly across all datasets, indicating dataset-dependent behavior.

### 3.3 Selection of important features

**Figure 6:**
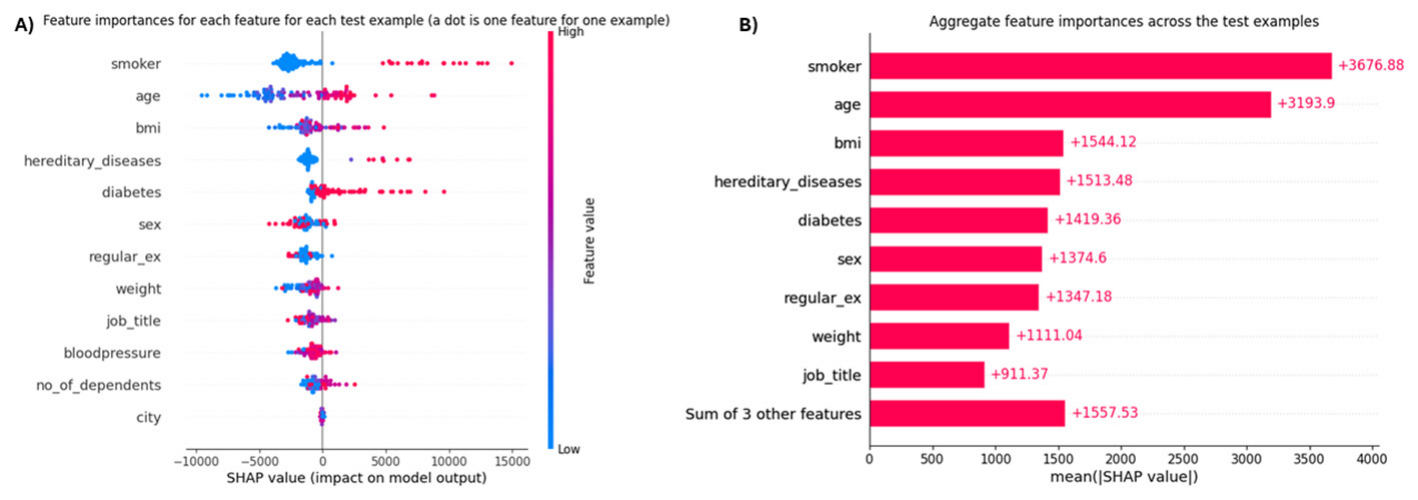
The SHAP summary plot and relative importance of each feature in the Tab-PFN model: **(A)** SHAP summary plot; **(B)** relative importance.

The beeswarm summary plot (Figure 6A) and the bar plot (Figure 6B) summarize the overall importance of each feature, as obtained through the SHAP-based interpretability analysis, and provide an effective method for quantifying the contribution of each input. It provides details on how various dataset features impact model output, as seen in SHAPley values combined with feature importance and impacts. The color gradient in the beeswarm summary plot indicates whether a feature's impact increased or decreased the predicted cost. Smoking status emerged as the most important feature and had the highest overall mean absolute SHAP value, reflecting its strongest contribution to the TabPFN model's predicted insurance costs. Features such as age and body mass index, and clinical variables such as hereditary diseases and diabetes showed moderate to high importance, while features such as regular exercise, sex, weight, blood pressure, job title, number of dependents, and city had a limited and minor influence on the prediction variability. The SHAP analysis provides deeper insights into the model's decision-making process, ensuring greater transparency in how insurance prices are predicted. It is important to note that SHAP values reflect each feature's marginal contribution to the model's predictions and should not be interpreted as evidence of causal effects. All interpretations presented here are associative in nature.

## 4 Discussion

This study investigated the performance of a recent Transformer-based foundation model, TabPFN, for predicting medical insurance claims and compared it with established tree-based models commonly used in tabular datasets. Previously, medical insurance prediction using tree-based machine learning methods has been extensively used (35, 41–43), nevertheless, these models often require extensive feature engineering and hyperparameter tuning. However, the advancements of AI models have impacted the insurance industry for automation, risk classification, insurance underwriting, Customer Lifetime Value (CLV) prediction, and fraudulent detection (22). They can better capture complex relationships in insurance data than linear models. Furthermore, the interpretability of these models is highly significant to insurance companies when advising customers on the most suitable health insurance plan based on their health records. The integration of SHAP with TabPFN in this study has shown that it is possible to achieve both high predictive performance and interpretability. The comparative results from our prediction showed that all the models achieved impressive outcomes, with the TabPFN model achieving the best *R*^2^ score of 0.9776. The model's performance and accuracy were further strengthened when tested across three other health insurance datasets, yielding *R*^2^ values of 0.882, 0.9998, and 0.817, respectively. The high Shapley value of the Smoker feature in our health insurance risk evaluation model underscores its pivotal role in capturing and quantifying health insurance risk.

Our analysis revealed that tabular foundation models outperform traditional ensemble methods and neural network designs specifically designed for tabular data. We evaluated TabPFN using various datasets and demonstrated the model's strength and ability to generalize in different insurance settings. This work contributes methodologically by demonstrating how in-context learning-based foundation models can be applied to actuarial prediction tasks without requiring extensive feature engineering or hyperparameter tuning. This marks a shift from the traditional workflow in insurance machine learning, which usually depends on iterative model optimization and adjustments specific to datasets. Finally, our study pushes the boundaries of explainable AI research in insurance by combining SHAP with the foundation model. While interpretability has been studied extensively for tree-based methods, this work demonstrates that high-performing foundation models can also be made transparent. This helps overcome a major obstacle to their use in regulated and risk-sensitive areas like insurance.

The novelty of this work lies in the application and systematic evaluation of a pretrained Transformer-based foundation model (TabPFN-2.5) within the health insurance prediction domain, demonstrating its superior performance and interpretability relative to established tree-based and neural network baselines without requiring task-specific training or hyperparameter optimization. This model will assist insurance companies in designing personalized, data-driven health insurance plans that align with individual customer profiles and anticipated future needs. The strong predictive performance of TabPFN-2.5, along with the inherent interpretability provided by SHAP-based explanations, can enable insurers to deploy these models with significantly less development effort, thereby reducing the need for labor-intensive parameter tuning and manual feature engineering.

Despite the significant contributions of the TabPFN has certain limitations. The model only deals with cross-sectional tabular datasets which leads to the losing of temporal dynamics in insurance claims. The model can be upgraded in future to handle larger datasets (44), longitudinal and time-series insurance data (45) and multi-modal data such as clinical records, wearable data, or textual claims descriptions, represents a promising direction for advancing predictive accuracy and risk assessment capabilities. An important limitation of this study is that all datasets used for evaluation including the three validation sets were sourced from Kaggle repositories and may share structural and distributional similarities. Consequently, conclusions about generalizability to real-world clinical or actuarial datasets should be made with caution. Future work should evaluate TabPFN on proprietary insurance datasets with diverse demographic and geographic distributions to establish broader external validity. Mean imputation was applied to the Age and BMI columns, which exhibited near-symmetric distributions (skewness within ±0.5); however, we acknowledge that this approach may modestly compress variance, and future work could assess sensitivity to alternative imputation strategies such as median or k-nearest neighbor imputation.

## 5 Conclusion

The integration of advanced AI models into health insurance represents a transformative shift in the industry, offering the potential for greater efficiency, cost savings, and improved patient care. The study investigates the recent Transformer Model (Tab-PFN) developed specifically for tabular datasets and for estimating health insurance prices. The model was superior to traditional machine learning models, bypassing manual feature engineering or architecture selection to predict health insurance claims directly. The model was evaluated using four health insurance datasets from the KAGGLE database, and the predicted results were explained using SHAP. Additionally, the TabPFN-2.5 has significantly reduced the previous size limitation of its earlier models. The results of this study will serve as a benchmark for health insurance organizations to deploy Tab-PFN in real time.

## Data Availability

The original contributions presented in the study are included in the article/Supplementary material, further inquiries can be directed to the corresponding author.
