# Supplementary material for: Evaluating TabPFN: a transformer-based foundation model for explainable health insurance claim prediction
Source: Front Public Health. 2026 Jun 26;14:1880390. doi: 10.3389/fpubh.2026.1880390 (PMC13352762; doi:10.3389/fpubh.2026.1880390)
Supplement: Supplementary file 1 [file Table_1.DOCX]

| **Characteristic** | **Primary Dataset** | **Validation Dataset 1** | **Validation Dataset 2** | **Validation Dataset 3** | **Notes** |
| --- | --- | --- | --- | --- | --- |
| Kaggle Source (Author) | Suresh Gupta | Mosap Abdel-Ghany | Toya King | Ugochukwu E. Orji | All datasets publicly available on Kaggle |
| Sample Size (after cleaning) | 13,90s4 | 1,338 | 1,332 | 986 | Primary dataset after removal of 1,096 duplicates |
| Number of Features | 13 | 7 | 11 | 11 | Feature counts exclude the target variable |
| Target Variable | Claim (USD) | Charges (USD) | Claim Amount (USD) | Charges (USD) | All targets are continuous regression outcomes (USD) |
| Shared Core Features | Age, Sex, BMI, Smoker, Dependents | Age, Sex, BMI, Smoker, Children/Dependents | Age, Sex, BMI, Smoker, Dependents | Age, Sex, BMI, Smoker, Dependents | Smoking status and BMI present in all four datasets |
| Additional Features | Weight, Blood Pressure, Diabetes, Hereditary Disease, Regular Exercise, Job Title, City | Region | Region, Insurance Type, Occupation | Region, Occupation, Exercise, Marital Status, Income | Primary datasets are richest in clinical and socioeconomic features |
| Missing Values | Age: 396; BMI: 956; Others: 0 | None reported | None reported | None reported | Mean imputation applied for Age and BMI in primary dataset (skewness within ±0.5) |
| Duplicate Rows Removed | 1,096 | 0 | 0 | 0 | Exact row duplicates removed prior to analysis |
| Categorical Encoding | One-hot encoding | One-hot encoding | One-hot encoding | One-hot encoding | Encoding applied within CV pipeline to prevent leakage |
| Target Distribution | Right-skewed (skewness = 1.48); mean ~$13,431 | Right-skewed; mean ~$13,270 | Near-linear structure; low variance; mean ~$8,900 | Right-skewed; mean ~$12,800 | Validation Dataset 2 exhibits low noise, explaining near-perfect R² = 0.9998 |
| Mean R² (TabPFN, Testing) | 0.9776 | 0.8821 | 0.9998 | 0.831 | Unusually high R² on Val. Dataset 2 attributed to homogeneous, low-noise structure |
| Potential Distribution Shift | Reference dataset | Fewer features; regional bias | Highly structured | Socioeconomic features differ | All datasets share smoking status as the dominant predictor |

**Table S1: Characteristics of All Four Datasets Used in This Study**
